# Establishment of Integrated Biobank for Precision Medicine and Personalized Healthcare: The Tohoku Medical Megabank Project

**DOI:** 10.31662/jmaj.2019-0014

**Published:** 2019-08-06

**Authors:** Nobuo Fuse, Mika Sakurai-Yageta, Fumiki Katsuoka, Inaho Danjoh, Ritsuko Shimizu, Gen Tamiya, Fuji Nagami, Hiroshi Kawame, Shinichi Higuchi, Kengo Kinoshita, Shigeo Kure, Masayuki Yamamoto

**Affiliations:** 1 Department of Integrative Genomics, Tohoku Medical Megabank Organization, Tohoku University, Sendai, Japan; 2 Department of Education and Training, Tohoku Medical Megabank Organization, Tohoku University, Sendai, Japan; 3 Department of Molecular Hematology, Graduate School of Medicine, Tohoku University, Sendai, Japan; 4 Statistical Genetics Team, RIKEN Center for Advanced Intelligence Project, Tokyo, Japan; 5 Department of Public Relations and Planning, Tohoku Medical Megabank Organization, Tohoku University, Sendai, Japan; 6 Department of Applied Information Sciences, Graduate School of Information Sciences, Tohoku University, Sendai, Japan; 7 Department of Pediatrics, Graduate School of Medicine, Tohoku University, Sendai, Japan; 8 Department of Medical Biochemistry, Graduate School of Medicine, Tohoku University, Sendai, Japan

**Keywords:** genomic medicine, personalized healthcare, precision medicine, Tohoku Medical Megabank Project, whole-genome reference panel, Japonica array, clinical sequencing

## Abstract

The Tohoku Medical Megabank (TMM) project was established to provide creative reconstruction of the Tohoku area that suffered from a huge earthquake and ensuing tsunami (the Great East Japan Earthquake, GEJE). TMM aims to establish two large-scale genome cohorts and an integrated biobank managing biospecimen and related information. It supports community medicine by establishing next-generation medical systems through a combination of the prospective genome cohort studies with a total of 150,000 participants and genomic medicine. The strategies for genome analyses in TMM are to develop an elaborate genome reference panel by means of high-fidelity Japanese whole-genome sequence, to design custom single nucleotide polymorphism (SNP) arrays based on the reference panel, and to obtain genotype data for all the TMM cohort participants subsequently. Disease-associated genomic information and omics data, including metabolomics and microbiome analysis, provide an essential platform for precision medicine and personalized healthcare (PHC). Ethical, legal, and social issues (ELSI) and education are important for implementing genomic medicine. The major considerations of ELSI regarding each participant of the cohort studies are the respect for the autonomy and the protection of privacies. Moreover, developing and provide human resources not only for the TMM project but also for the social implementation of precision medicine and PHC is required. We started a pilot study of the return of genomic results for familial hypercholesterolemia (FH) as a target disease. TMM aims to establish solid platforms that support precision medicine and PHC based on the genomic and omics information and environmental and lifestyle factors of the individuals, which is one of the most advanced medical care beyond the evidenced-based medicine in the near future.

## Introduction

**Figure 1.**
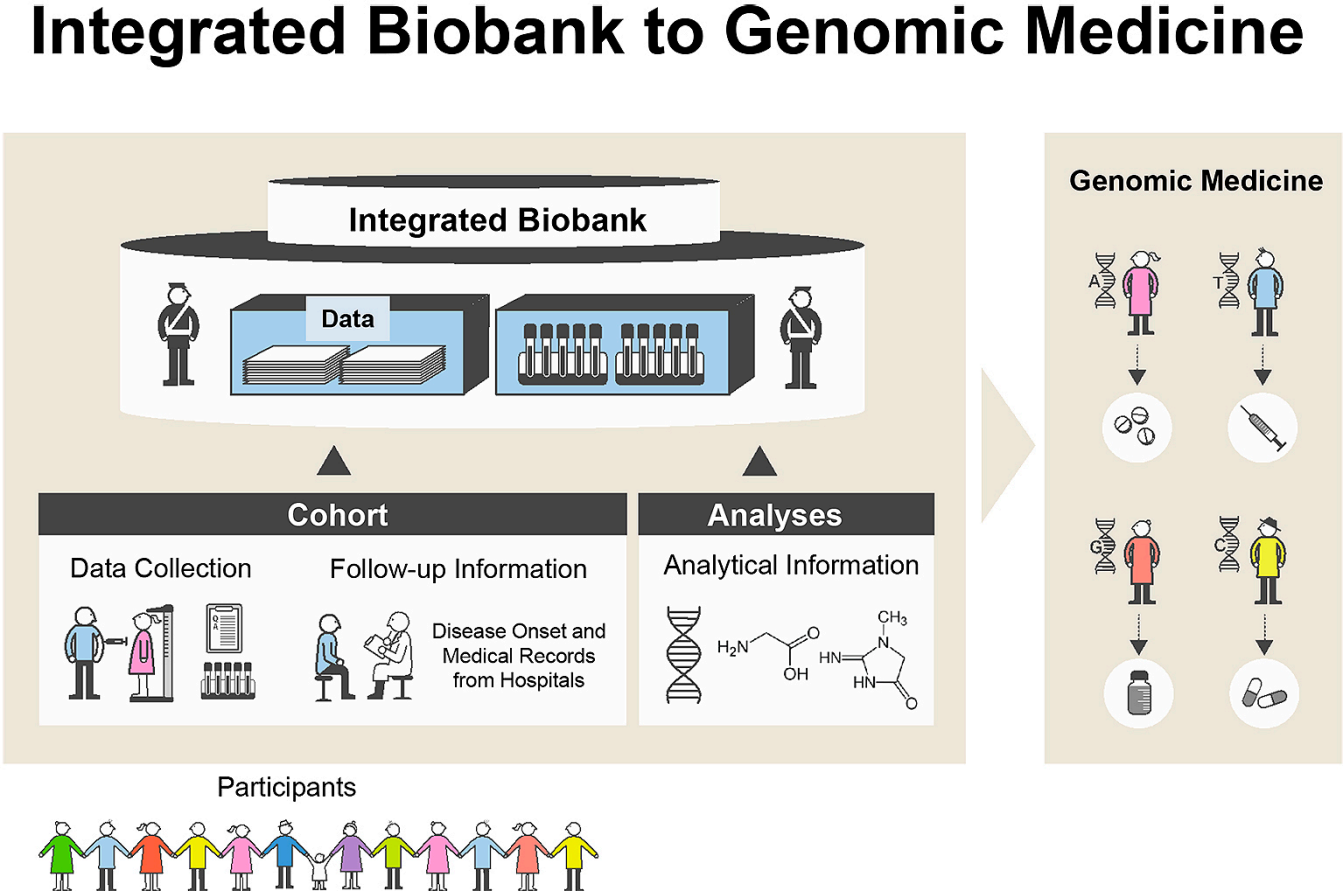
TMM establishes “integrated biobank” consisting of biospecimens, information of cohort studies, and genome-omics analyses. The integrated biobank is an important research infrastructure for implementing genomic medicine.

A huge earthquake (the Great East Japan Earthquake, GEJE) with tremendous tsunami waves damaged wide areas of the northeastern coast of Japan on March 11, 2011 ^(1)^. Bad circumstances, imbalanced nutrition ^(2)^, and the lack of daily activities influenced on people who suffered from the earthquake ^(3)^. To revive medical services in those damaged regions, we proposed the Tohoku Medical Megabank (TMM) Project to establish next-generation medical systems through a combination of the prospective genome cohort studies with 150,000 participants and medical genetics in Miyagi and Iwate Prefectures. To conduct the TMM Project in the two prefectures, the Tohoku Medical Megabank Organization (ToMMo) was established at Tohoku University in Miyagi Prefecture, and the Iwate Tohoku Medical Megabank Organization (IMM) was established at Iwate Medical University in Iwate Prefecture. We constructed three platforms: *i*) biobank, *ii*) two types of prospective genome cohorts, and *iii*) genome and omics analysis (Figure 1). The purpose of our project is to develop the fundamental infrastructure consisting of genome and cohort information for the establishment of precision medicine and personalized healthcare (PHC) in Japan.

## Establishment of Two TMM Cohorts

**Figure 2.**
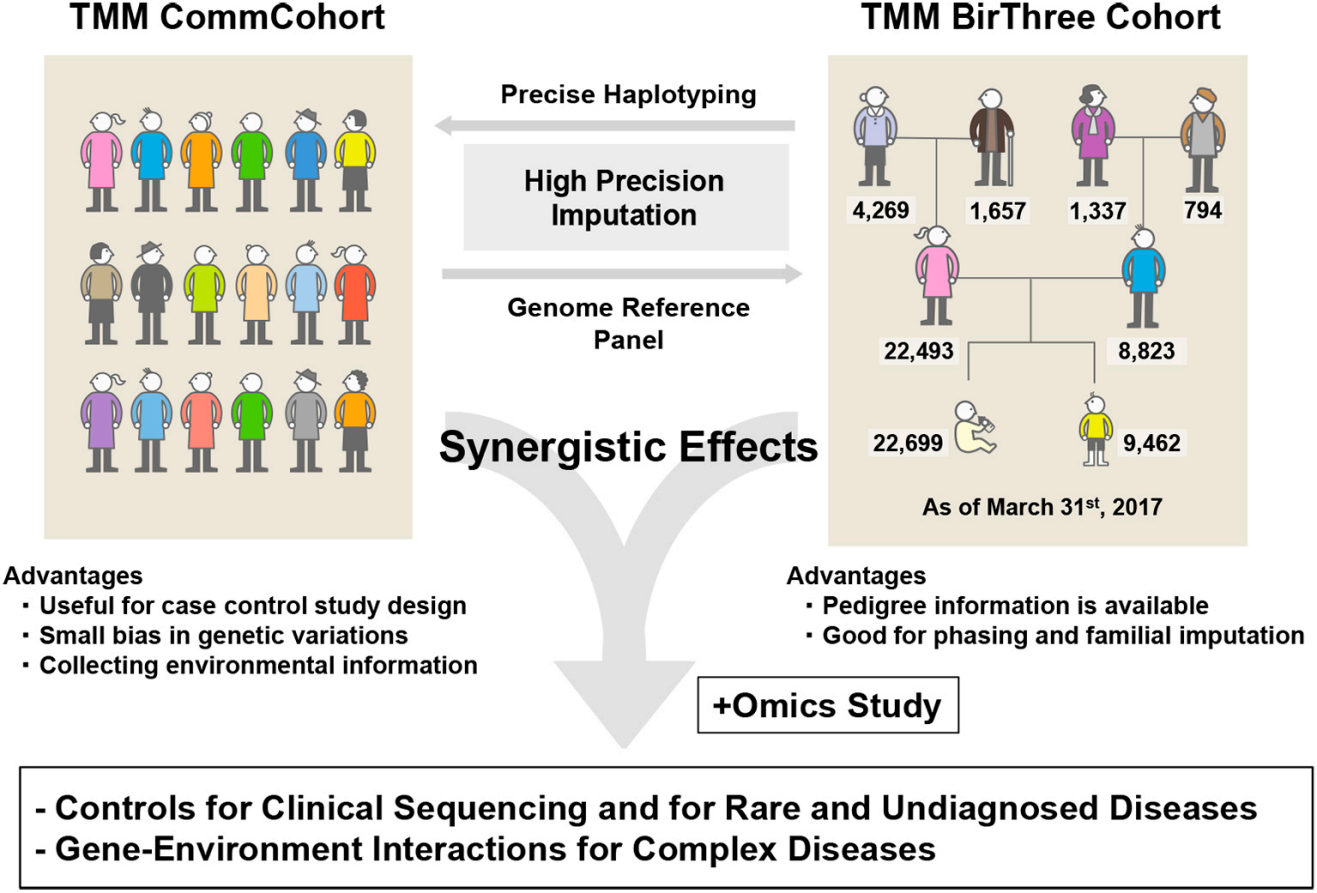
TMM establishes two cohorts, TMM CommCohort and TMM BirThree Cohort. Taking advantages of these cohorts and their synergistic effects, we provide the reference panel as control data for clinical diagnosis and establish risk prediction scores of complex diseases by gene-environment interaction studies.

TMM has been working to establish two cohort studies ^(4)^ (Figure 2). One is the TMM community-based cohort study (TMM CommCohort Study), in which a total 84,073 participants (52,212 and 31,861 from Miyagi and Iwate, respectively), 20 years or older (mean age, 61 years), were recruited by March 2016. The other is the TMM Birth and Three-Generation Cohort Study (TMM BirThree Cohort Study), which included pregnant mothers, their husbands (or partners), their parents, the newborn babies, and their elder siblings. In the TMM BirThree Cohort Study, a total of 73,500 participants were recruited from the obstetrics hospitals and clinics in the Miyagi Prefecture by July 2017 from 22,493 families. Through the TMM BirThree Cohort Study, we are hoping to collect longitudinal data from the pregnancy and postnatal periods to adulthood.

To conduct a smooth recruitment of residents for the TMM CommCohort Study and TMM BirThree Cohort Study, we established seven local community support centers in Miyagi Prefecture and four satellites in Iwate Prefecture. We performed a number of examinations, such as central aortic blood pressure, body composition, bone density, grip strength, leg strength, oral health, respiratory function, hearing, and eye examinations and carotid ultrasound imaging. Moreover, in the TMM project, an “integrated biobank” consisting of biospecimens, health and clinical information, and genome-omics data for the research infrastructure was established.

PHC is the concept in which individual health should be managed by means of the individual’s specific characteristics. PHC actively utilizes the individual’s genomic and omics profiles by exploiting appropriate methods and interventions to prevent various acquired diseases. In addition, genomic medicine is an emerging medical discipline that utilizes genome information of individuals for clinical care. The use of genomic medicine is now expanding to the prediction of the safety and effectiveness of medical treatments and risk assessments of each individual. Precision medicine and PHC are becoming two of the most advanced but feasible forms of medical care with multiple approaches to personal care. Precision medicine and PHC have the potential to detect diseases at earlier stage and as such easily make the treatment effective. Full implementation of precision medicine and PHC should involve risk assessment, prevention, detection, diagnosis, treatment, and management of diseases. Because TMM project is based on two prospective genome cohort studies, we are focusing especially on risk assessment and prevention.

Theoretically, a prospective cohort study treats all diseases that occur in the participants, who are ostensibly healthy people. However, our major focus is on the common diseases that involve an interaction between genetic and environmental factors. Therefore, we informed the cohort participants of the major diseases we are especially focusing on: cancers, cardiovascular disorders, diabetes, mental diseases (PTSD and depression), dementia, and respiratory diseases, such as asthma and chronic obstructive pulmonary disease. In the TMM BirThree Cohort, the target diseases also include atopic dermatitis, autism, and low birth weight of babies and preeclampsia of mothers ^(4)^.

To firmly establish PHC, the association between disease phenotype, environmental factors, and genotype-omics data must be deeply investigated. In the case of Mendelian diseases, highly penetrant disease-responsible variants can be easily identified. In contrast, in the case of common and multifactorial diseases, a large number of common and rare variants associated with such diseases must be sought for the risk estimation. A number of single nucleotide polymorphisms (SNPs), which are associated with many human traits or diseases, were already reported. However, these disease-associated SNPs cannot sufficiently explain the heritability of disease susceptibility. The discrepancies between observed heritability and the contributions to disease susceptibility by the disease-associated SNPs have been referred to as “missing heritability” ^(5)^.

Nowadays, the advent of next-generation sequencing and SNP array analysis with genotype imputation enabled us to acquire information of all variants from a population. In this regard, TMM has been focusing on providing the essential infrastructure, which is a large set of genome variation data of the general residents. This genome reference panel would contribute as control data for collaborative association studies. Thus, TMM provides an analytical platform that medical doctors and researchers are able to perform collaborative studies by using genome and medical data under secure protection of privacy and personal information.

The project protocols were reviewed and approved by the Ethics Committee of Tohoku University Graduate School of Medicine and by the Ethics Committee of Iwate Medical University, and written informed consent was obtained from all of the participants.

## Strategy of Genome and Omics Analyses on Genome Cohorts

**Figure 3.**
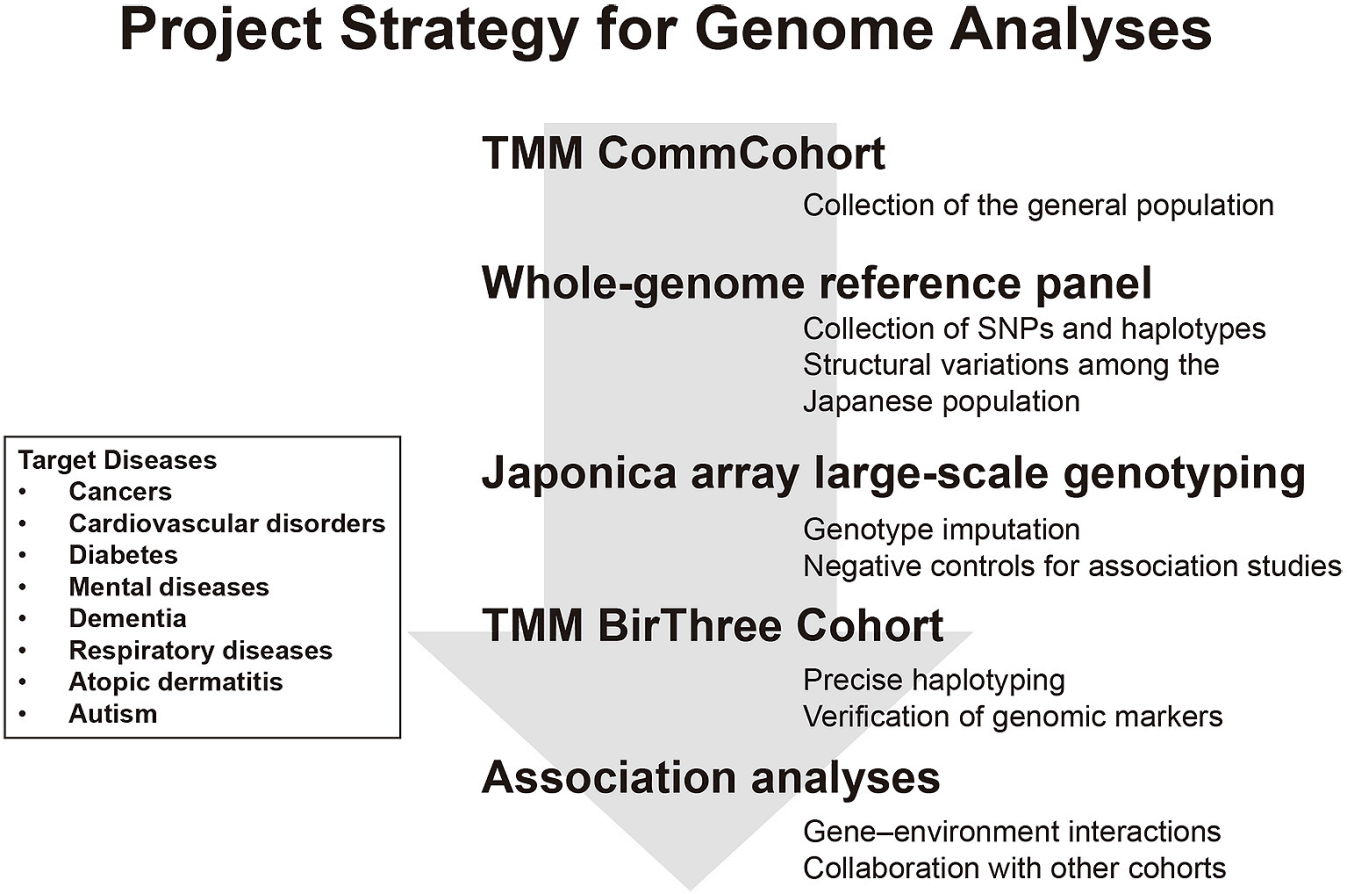
Our strategy of genome analyses and target diseases. A whole-genome reference panel is developed by using TMM CommCohort and applied for genotype imputation subsequent to large-scale genotyping by Japonica array™. The TMM BirThree Cohort is utilized for the collection of accurate haplotype information.

We constructed two kinds of cohorts, i.e.,**TMM CommCohort and TMM BirThree Cohort. Our strategy for the genome and omics analyses intends to maximize the benefits of these two different cohorts, which recruited healthy volunteers. We planned to construct reference panels of Japanese genome and omics first mainly using the TMM CommCohort Study. Based on the information derived from the reference panels, we planned to prepare Japonica array for large-scale genotyping of the Japanese population and, subsequently, to utilize the TMM BirThree Cohort for verification of the results of genome analyses conducted using the Japonica array. The TMM BirThree Cohort may also be useful for the construction of precise haplotype collections capitalizing their familial information. Thus, while these two cohorts are managed independently, the combination of the two types of cohorts in the genome and omics analyses will be effective in revealing important mechanistic insights of complex diseases caused by gene-environment interactions (Figure 3).

Based on the above considerations, we first selected approximately 1,000 participants from the TMM CommCohort and performed whole-genome sequencing (WGS) analysis. To clarify the genetic variations in the Japanese population, we constructed a Japanese whole-genome reference panel based on the WGS data ^(6)^. As will be described precisely below, we then developed a custom SNP array specially designed for the Japanese population to expedite collection of genotype data ^(7)^. Indeed, we are expediting the collection of genomic information of 150,000 participants by using the SNP array, as genome data of the TMM BirThree Cohort will be significant to improve accuracy of haplotypes. It should be noted that accurate haplotype collection could be used for precise genotype imputations with the SNP array.

As for the omics analyses, we conducted transcriptome, epigenome, proteome, metabolome, and metagenome analyses. By using these omics and other data, we are trying to clarify the gene-environment interactions within the Japanese population and accumulate solid lines of evidence for the realization of future medicine in Japan ^(8)^. These fundamental strategies of genome-omics analyses share certain similarity with those of deCODE study in Iceland, as well as other modern genome cohort studies, such as UK Biobank and Finland Biobank. Meanwhile, it should be noted that the TMM project is unique as it is operating two closely related cohorts in an elaborate combination and also conducting the birth and three-generation cohort successfully, even though similar birth and three-generation cohort attempts in the United States and the United Kingdom were cancelled as a result of the shortage of participants ^(9)^. Since the inception of the TMM project in 2012, we have been steadily analyzing the participants’ genome and omics following the strategy summarized here. For more details on the genome-omics analyses of our project, please see Yasuda et al. ^(10)^ and Koshiba et al. ^(11), (12)^.

## Genotyping Using Japonica Array™

To establish the precision medicine, it is mandatory to collect precise genome data from all cohort participants. In this regard, although WGS gives rise to detailed information of each genome, its use for the entire cohort samples is not realistic, as it is too expensive. To solve this contradiction, a microarray approach coupled with a genotype imputation method is often employed in various genome cohorts worldwide ^(13)^. Polymorphic genome reference panels with haplotype phasing are necessary for the genotype imputation of microarray data. As patterns of linkage disequilibrium (LD) block are distinct among populations ^(14), (15)^, imputation accuracy heavily depends on the quality of both reference panel and microarray data. To obtain highly accurate performance of the imputation, the genome reference panel constructed with genome data from thousands of individuals and genotyping array designed to include polymorphic markers reflecting the LD block pattern are necessary.

We started the development of Japanese-specific full custom array, referred to as Japonica Array™ (JPA) in 2013 ^(7)^. Until the end of 2018, we produced three full custom arrays, JPAv1, JPAv2, and JPAv3. Currently, JPAv2 is mainly used for the data acquisition of TMM cohort samples (Table 1). JPAv1 and JPAv2 have already been placed on the market by Toshiba. JPAv3 is focused on disease-related SNPs identified in the Japanese population as well as tag SNPs.

**Table 1.** Contents of Japonica Array^TM^ v2.

Category	Number of SNPs
Tag SNPs (including X chromosome)	632,186
Pharmacogenomic markers (ADME)	2,020
Y chromosome	606
Mitochondria	104
NHGRI GWAS catalog	11,171
HLA	6,914
Untagged functional SNPs	3,990
Total	659,328

We collected SNP data from our cohort participants by using JPAv1 and JPAv2 that are optimized for genotyping of the Japanese population. Genotyping data, in 2018 fiscal year, were obtained from >68,000 participants. Mainly using JPAv2, we will complete the collection of microarray genotype data from 114,000 participants by the end of March 2019.

We set up analytical pipelines consisting of wet laboratory- and informatics-based processes in ToMMo, and most of the genotyping assays were performed in the ToMMo facility. Our wet laboratory equips Laboratory Information Management System (LIMS) for quality control and has the capacity to acquire JPA data for more than 6,000 samples per month. The acquired data is automatically transferred into supercomputer and imputed by using the 3.5KJPNv2 reference panel subsequent to multiple steps of genotyping and quality control analyses. At present, imputed dataset consisting of individual-level genotype data is released on approximately 32,000 TMM participants, including genomic data obtained by SNP arrays other than JPA.

We are planning to complete genotyping of all participants (more than 150,000) by March 2021 and to release full dataset after imputation in line with our data-sharing policy. In addition, we are planning to share our analytical pipelines for our collaborators who have various case samples. These approaches would promote the study of gene-environment interaction and the development of disease risk scores, bringing about the realization of genomic medicine.

## Risk Analysis Based on Genome Information

A true set of genetic and environmental factors conferring the risk of various complex diseases is valuable for the prediction of disease onset or prognosis. However, disease risk predictions are still in their infancy, chiefly due to uncaptured genetic components, biased environmental measures, complex nonadditive combinatorial effects, insufficient experimental/statistical methods, and so on ^(16)^. Large-scale prospective genomic cohort with biobank, such as TMM, will provide an opportunity to overcome such limitations in the disease risk predictions ^(5)^. From identical populations in TMM, one can obtain a more comprehensive assessment of the full range of human genetic variation and less-biased environmental measurements ^(17)^. Unlike scores based on any single risk factor, some sophisticated multivariate models including ours ^(18), (19)^ are now beginning to yield more accurate scores than before even under the large-p small-n conditions in standard genome-wide association studies (GWASs).

One of our methods, Iwate polygenic model (iPGM), efficiently utilizes GWAS data along with environmental factors using a framework of linear mixed model ^(19)^. Another method that we developed, smooth-threshold multivariate genetic prediction (STMGP), is a statistical machine-learning method based on an analogous framework of ridge regression that gets around p >> n problem from yielding the best prediction performance (or area under the curve, AUC) among known methods in the other public cohorts ^(18)^.

Using these approaches as core algorithms coupled with a large-scale genetic and environmental data from TMM, we will carry out our disease risk predictions in Miyagi and Iwate. We will then expand the risk prediction to the other areas of Japan. Our effort could overcome the missing heritability problem to realize disease risk predictions by incorporating a comprehensive set of disease-contributing factors: additive, nonadditive genetic factors, environmental factors, and their interactions. Showing very good agreement with our approach toward these improvements in data collection and risk analyses, polygenic risk scores (PRSs) generated by the DNA microarray analyses and other clinical examinations have been highly expected to predict risks of multifactorial diseases. We theorize that the time to exploit genome analysis data through PRSs tests and to predict disease risks for the complex diseases in clinic should come soon ^(20)^.

## Metagenomic Analysis

We are continuously exposed to environmental microbial species as well as environmental chemicals, pollutions, and ultraviolet. Metagenomics is a direct genetic analysis of the genomes of microorganisms that reside in an environmental sample. Recent advances in high-throughput genome-sequencing technique and in bioinformatics allow us to verify precise taxonomic classification in the microbiome communities. Experimental lines of evidence were gathered to show that the patterns of human microbiomes are different between healthy people and patients with specific diseases. Thus, characterizations of the human microbiomes, especially symbiotic microbiomes residing in our body parts, and analyses of the roles that they play in health and disease conditions are emerging as a growing scientific interest.

We developed an oral metagenomics as one of the pipelines. Characterizations of multiple microbiomes are expected to contribute to health and disease monitoring. Multiple microorganisms of different species inhabit the plaque in the oral cavity. Buildup of plaque is an essential characteristic of oral infectious diseases ^(21)^, such as periodontal disease and dental caries, which affect nearly all ages and all ethnicity of people worldwide. Certain species of oral microbiomes are thought to play important roles in modulating host response not only in the oral cavity but also in the whole body, by acting as pathogens and/or changing metabolic pathways ^(22), (23)^. Comprehensive analysis by metagenomics together with host genomics and metabolomics is an important strategy for public health surveillance and risk prediction.

We collected plaque and saliva from more than 25,000 participants and analyzed their microbial profiles using the 16S ribosomal RNA gene high-throughput sequencing. We are planning to investigate not only the associations of oral microbial profiles with host phenotypes but also the cross relationships of multiple phenotypes accumulating in the TMM project. We theorize that this comprehensive approach brings about the understanding as to what is more influential to the onset of diseases among environmental, lifestyle, and genetic factors.

## Ethical, Legal, and Social Issues (ELSI)

The TMM project is a huge, complex, and multidirectional project. At the planning stage of the project, the adequacy of the genome cohort project as a reconstruction program for the damage caused by the GEJE was widely discussed. The frameworks of the project, especially those of the cohort studies, were arranged to maximize the benefit of the community located in or near the devastated area. Therefore, results of the baseline cohort studies are returned to each participant. In addition, statistical results of the studies are also shared with the municipalities to contribute to the improvement of the people’s health in the community.

One of the major ELSI considerations regarding the participants of the cohort studies is to respect the autonomy and to protect their privacies. During the recruitment, we took care of each resident’s decision to participate or not in the cohort studies independently. For this reason, we have separated the recruitment interviews not for clinical but for research setting. All informed consents (IC) were obtained by our specially trained genome medical research coordinators (GMRCs), many of whom are also certified by the Japan Society of Human Genetics (JSHG). Information of the cohort studies is de-identified and stored in our supercomputer, which is independent from the other networks. Genome sequence information is treated carefully with high-level security; all analyses using the genome sequence data are operated in the TMM supercomputer, and only analytical results without personal identification are taken out from the security area in the supercomputer.

The TMM project is the first large-scale biobank in Japan, which is set up on population cohorts and declares the sample and data sharing. As an integrated biobank, the TMM biobank is under the data- and sample-sharing policy that is not so familiar to the academia and industries. We distribute data and samples without insisting the intellectual property from us.

The TMM biobank proposes a number of novel rules as well as governing structures for better system operation and compliance with the ELSI requirements. Now, our challenge moves on to the social implementation of precision medicine, which we theorize has many ELSI concerns, especially at the interface of community medicine. To this end, we are planning to make tight relations with community medical professionals.

## Education and Training

Human resource development is necessary for the implementation of TMM project consisting of multiple activities, including community medical support, large-scale cohort study, and genome and omics analyses. As described above, our GMRCs obtained IC from all of the cohort participants via face-to-face explanation and worked for the baseline assessment. Nearly 300 persons were cumulatively authorized as ToMMo GMRCs by an original training and education program, which offers more intensive training than that for GMRC certified by JSHG. Approximately half of them are still playing important roles for close communication with participants in the TMM repeat assessment center-based survey during the second phase of the TMM project.

Considering the social implementation of genomic medicine, Graduate School of Medicine at Tohoku University and Iwate Medical University, our close collaborators, have now opened genetic counseling education programs. Certificated genetic counselors and medical geneticists are working on a pilot study that returns genome analysis results of a monogenic disorder. As described above, upon the arrival of the days in which we take an advantage of PRSs or risk assessment in the clinic, we need to consider by whom and how the genetic risks of common diseases are returned for realizing precision medicine and PHC, which is the main goal of the TMM project.

In addition, it has been known that in Japan, experts of bioinformatics are mainly educated in graduate schools, whereas biobank managers and specialists of medical informatics are educated almost exclusively in on-the-job trainings. In anticipation of making more progress of genomic medicine, comprehensive education by taking advantage of TMM facilities and resources could be one of the ways to develop specialists in genomic medicine and make them available throughout Japan. In this way, it is required to develop and provide human resources not only for the TMM project but also for the social implementation of precision medicine.

## Return of Individual Genomic Results

Since the completion of the first human genome sequencing and subsequent emerging systematic catalog of human genetic variation ^(15)^, there has been a growing expectation that the association between variations and disease susceptibility will contribute to precision medicine and PHC. We focused on three types of genetic variants that cause various disorders: rare variants in genes responsible for Mendelian diseases, variants that could modulate responses to drugs, and variants associated with the risk of complex or common diseases, including diabetes mellitus, hypertension, coronary diseases. As WGS becomes available in various research settings, obtaining individual genome information is now feasible for the application of genomics to the clinical care.

In 2013, the American College of Medical Genetics and Genomics (ACMG) published a guideline of incidental findings in exome and genome sequencing to make recommendations about responsible management of secondary findings when patients undergo exome or genome sequencing in the clinical setting ^(24), (25)^. In the updated paper, the minimum list of the secondary findings includes 59 medically actionable genes that are recommended for return in clinical genomic sequencing.

One of the missions of our genomic cohort studies is to immediately provide the precision medicine and PHC for our cohort participants and create experience of return of individual genomic results from the research setting, which has not been systematically analyzed or operated. After extensive discussion and literature review ^(26)^, we choose familial hypercholesterolemia (FH) as a target disease for the first pilot study of the return of genomic testing to the individual participants. FH is an actionable disease, listed in the ACMG guideline, and identifying those with FH through genomic analysis has allowed early treatment. Return of genomic results could expect outcomes such as facilitation of diagnoses, initiation of therapies at earlier ages, and more accurate risk estimation, especially for family members.

This pilot study included approximately 200 cohort participants, who finally gave informed consent after the “genetic workshop,” which contains the basic genetics, unique features of genetic information, and medical facts of FH. About 10% of the participants were positive for pathogenic or likely pathogenic SNPs in *LDLR*,* PCSK9*, and *APOB* genes. The return of the individual results was done face-to-face by clinical geneticists or certified genetic counselors. Now, we are preparing the next pilot studies regarding pharmacogenomics information and low-penetrance and adult-onset diseases, such as hereditary cancer syndrome, i.e., hereditary breast and ovarian cancer (HBOC) and/or Lynch syndrome.

## Examples of Utilizing Our Genome Data in Clinical Studies

### 1. Sequencing for rare and undiagnosed diseases

Several patients are suffering from inherited rare diseases and seeking diagnosis. To save such patients from the so-called diagnostic odyssey, clinical whole-exome sequencing (WES) or WGS was applied to identify variants causing diseases. WES or WGS was successfully applied in clinical settings owing to the decrease in sequencing cost and the improvement of sequencing technologies. As part of such efforts, the Initiative on Rare and Undiagnosed Diseases (IRUD) project is ongoing in Japan led by the Japan Agency for Medical Research and Development (AMED) ^(27)^.

It is generally difficult to identify casual genes by sequencing only the proband, because in addition to causal variants, patients possess millions of variants irrelevant to diseases. To overcome such difficulties, it is ideal to sequence not only the probands but also their close relatives, such as parents who do not develop symptoms. Variants shared with their relatives are likely unrelated. However, it is not always possible to obtain such samples. In addition, sequencing of the relatives often results in vain, because enormous candidate variants remain even after excluding the shared variants. In such cases, ToMMo’s Japanese genome reference panel plays important roles ^(6), (10)^.

Our genome reference panel is based on whole-genome sequence data of the general population in Japan ^(10)^. As a result, variants cataloged in our panel are less likely to cause severe diseases. Based on this idea, many IRUD projects have been taking advantage of our reference panel to interpret their sequencing data. Information on variant frequencies is also critical to judge whether variants are benign, and a larger number of reference panel provides more accurate frequencies for rare variants. Thus, we have been updating our reference panel from 1KJPN (1,092 samples), 2KJPN (2,049 samples), 3.5KJPN (3,554 samples), to 3.5KJPNv2 (3,552 samples due to two consent withdrawals). It should be noted that, due to technical limitations of short-read sequencing, the current reference panels do not cover large structural variants (SVs). We are expecting that long-read sequencing, which will be practical in the near future, will allow us to construct more comprehensive reference panels including SVs.

### 2. Cancer clinical sequencing

Clinical sequencing is now widely adopted as advanced medical care of cancer treatment. Information on somatic variants that contribute to tumorigenesis and tumor malignancy is valuable to determine treatment policies, such as chemotherapy. However, compared with germline variants, sequencing of cancer somatic mutations is not that easy. First, it is practically difficult to extract tumor samples without contamination of normal tissues. This is especially the case for metastatic lesion. Second, tumors often get diversification through its development, leading to genetic heterogeneity.

For these reasons, much higher sequencing coverage (depth) is necessary for cancer clinical sequencing. In general, ×30 (WGS) or ×50-100 (WES) sequencing depth is necessary for germline variants. On the other hand, ×100 to ×1,000 sequencing depth is necessary for cancer clinical sequencing, depending on its purposes ^(28)^. Therefore, targeted sequencing methods, such as panel sequencing focusing on cancer-related genes, are often adopted. Interestingly, it has been reported that the so-called mutation load, the number of nonsynonymous mutations, can be utilized to predict the responsiveness to cancer immunotherapy ^(29)^. In such cases, WES or WGS is adopted to estimate the accurate mutation load by analyzing comprehensive datasets of somatic mutations.

Examination of germline mutations is also important in cancer genomics, because 5% to 10% of cancers are believed to be related to inherited variants in tumor suppressor genes, such as cell cycle-related genes and DNA repair genes ^(30)^. To estimate the frequency of such cancer risk variants, ToMMo’s reference panel will be helpful. For instance, we previously summarized the frequencies of pathological variants in the 2KJPN, for which the ACMG recommends returning of the results ^(31)^. According to this report, the 2KJPN reference panel includes five reported pathogenic variants in *BRCA1* and *BRCA2* genes whose germline mutations predispose to breast and ovarian cancer. The data also allows us to estimate the population frequencies of these susceptible variants. In this way, the reference panel of the general population is a powerful tool to obtain the fundamental knowledge necessary for genomic medicine.

## Conclusions

To realize precision medicine and PHC, integration of the genomic and omics data with lifestyle data is indispensable for the improvement of disease risk assessment and understanding of the biological processes of diseases for prevention, early diagnosis, and proper managements. We believe that prospective cohort studies in the TMM project will provide elaborate genomic and omics data platforms that actualize the genomic medicine. In the 21st century, many scientists pay great attention to precision medicine and PHC. The goals of our integrated biobank are to promote healthy lifestyles and to prevent and develop treatment methods suitable for each person’s constitution.

## Article Information

### Conflicts of Interest

None

### Sources of Funding

This work was supported in part by Tohoku Medical Megabank Project from the Ministry of Education, Culture, Sports, Science and Technology (MEXT) and AMED (grant numbers JP18km0105001, JP18km0105002), Platform Program for Promotion of Genome Medicine (P3GM; grant number JP18km0405001), Advanced Genome Research and Bioinformatics Study to Facilitate Medical Innovation in P3GM (GRIFIN; grant number JP18km0405203), and Center of Innovation Program from Japan Science and Technology Agency (JST).

### Acknowledgement

We would like to thank all the volunteers who participated in this study. We would also like to acknowledge all those associated with the study, especially Ms. Miho Kuriki for creation of artwork. The member list is available at the following website: http://www.megabank.tohoku.ac.jp/english/a181201/. We would also like to thank the Iwate Medical University Iwate Tohoku Medical Megabank Organization (IMM) for collaboration. 

### Author Contributions

Nobuo Fuse and Mika Sakurai-Yageta contributed equally to this work.

### Approval by Ethical Committee

The project protocols were reviewed and approved by the Ethics Committee of Tohoku University Graduate School of Medicine and by the Ethics Committee of Iwate Medical University.
